# Supplementary material for: Targeting ADAR stability: a novel approach to combat glioblastoma
Source: Front Oncol. 2026 Aug 6;16:1827793. doi: 10.3389/fonc.2026.1827793 (PMC13489870; doi:10.3389/fonc.2026.1827793)

Correlation coefficient = 2.52e−02; P−value = 5.68e−01

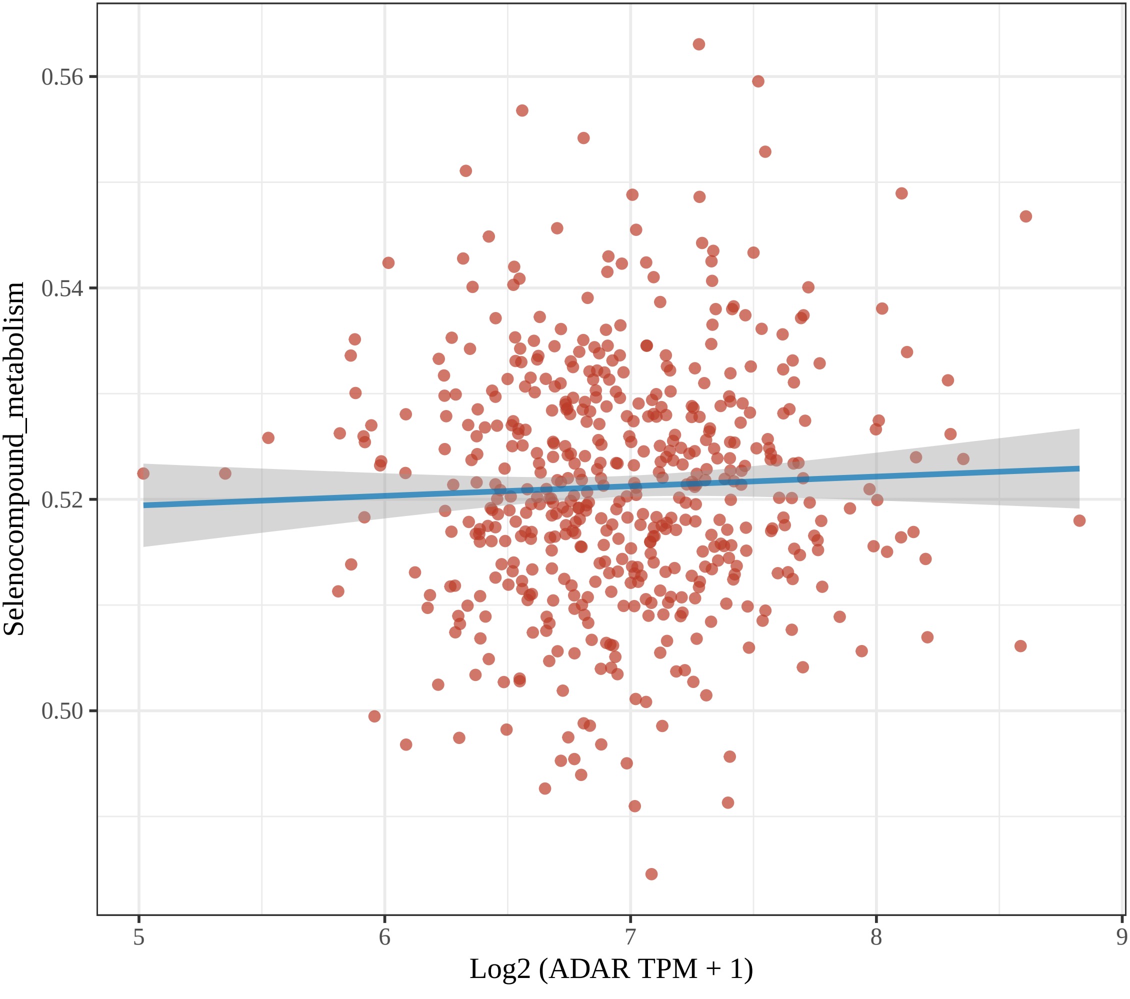

Supplement: Supplementary file 1 [file SupplementaryFile1.zip › Supplementary file 1/Selenocompound_metabolism.pdf]
